# Solution processed multi-layered thin films of Ge_20_Sb_5_S_75_ and Ge_20_Sb_5_Se_75_ chalcogenide glasses

**DOI:** 10.1038/s41598-023-43772-w

**Published:** 2023-10-03

**Authors:** Jiri Jemelka, Karel Palka, Petr Janicek, Stanislav Slang, Jiri Jancalek, Michal Kurka, Miroslav Vlcek

**Affiliations:** 1 Department of General and Inorganic Chemistry, Faculty of Chemical Technology, University of Pardubice, Studentska 95, 53210 Pardubice, Czech Republic; 2 Faculty of Chemical Technology, Center of Materials and Nanotechnologies, University of Pardubice, Studentska 95, 53210 Pardubice, Czech Republic; 3 Faculty of Chemical Technology, Institute of Applied Physics and Mathematics, University of Pardubice, Studentska 95, 53210 Pardubice, Czech Republic

**Keywords:** Materials for optics, Design, synthesis and processing, Optical materials and structures

## Abstract

Solution processed non-toxic Ge_20_Sb_5_Se_75_ chalcogenide glass thin films were deposited using spin-coating method from n-propylamine—methanol solvent mixture in specular optical quality. Optical properties, composition, structure, and chemical resistance were studied in dependence on the annealing temperature. Significant increase of refractive index and chemical resistance caused by thermoinduced structural polymerization and release of organic residua were observed. The high chemical resistance of hard-baked thin films allowed repeated direct depositions by spin-coating, increasing total thickness. Multilayered thin films of amorphous Ge_20_Sb_5_Se_75_ and Ge_20_Sb_5_S_75_ were also successfully prepared by direct deposition for the first time. Solution based deposition of non-toxic Ge_20_Sb_5_Se_75_ thin films in specular optical quality significantly widens the applicability of solution processed chalcogenide glass thin films. Moreover, solution based direct deposition of different glasses on hard-baked thin films opens the way to simple and cost-effective preparation of more sophisticated optical elements (e.g. beam splitters, photonic mirrors).

## Introduction

Chalcogenide glasses are promising semiconducting materials intensively studied for many decades mostly for their optical properties^1–4^. Chalcogen of the glass (S, Se or Te) determines the optical properties of the material. Heavier chalcogen provides glasses with significantly higher refractive index and lower band gap^5^. The amorphous character of these materials offers large compositional ranges in which chalcogenide glasses can be prepared, which makes them impeccable materials for tailoring of optical properties^6^.

Applications of chalcogenide glasses often require material in a thin film form of various thicknesses. Solution based deposition techniques are lately gaining attention as a chemical counterpart to traditionally used physical vapor deposition methods such as thermal evaporation (the most frequently used), sputtering, or laser ablation^3^. Acidic character of chalcogenide glasses allows the dissolution of source bulk glass in volatile organic bases (usually amines or amine-based solvent mixtures), providing a solution of the glass (an “ink”) which can be deposited by a number of coating and printing methods forming a thin film of the solution solidifying after the solvent evaporation^7,8^. The most frequently used deposition technique on a laboratory scale is spin-coating^9,10^. Other deposition methods have been successfully tested for larger scale thin films depositions as well (e.g. dip-coating^11,12^, electrospray^13^, spiral-bar coating^14^). In comparison with physical vapor deposition techniques, solution based deposition methods do not require expensive high vacuum equipment. For this reason, solution based methods are significantly cheaper (both initial investments and running costs), which makes them ideal for low cost mass production in many applications where flawless quality of the thin films is not needed.

Solution based process of chalcogenide thin films deposition offers a significant advantage in comparison to physical vapor deposition methods—an intermediate step in the solution state. The source solution of the glass for thin film deposition can be broadly modified in the composition and structure, which directly influences composition and the structure of final thin film. Our recent publications showed possibility to alter the composition of the source solution by pure elements (S and Se) in wide compositional range^15,16^, which allows to prepare thin film of various compositions without the need to synthesize each source bulk glass separately. This significantly simplifies the film preparation process and widens the possibilities of work with few commercially available glasses, even for workplaces without the equipment for bulk glass synthesis. The doping of the source glass solution by silver has also been intensively studied^17,18^ due to significant changes in physical and chemical properties, which silver induces when introduced to the glass structure^19,20^. Furthermore, solutions of different chalcogenide glasses can often be mixed together, providing a source solution of complex composition from simple (binary or ternary) glasses^21,22^. The structure of the prepared thin film can be influenced by the introduction of nanostructures of different materials (e.g. quantum dots, carbon nanotubes, etc.) into the source glass solution^23–25^. This process allows to bring in completely new characteristics to the thin film, such as photoluminescence^26^.

Advanced optical applications often require multi-layered thin films prepared from materials with diverse optical properties (e.g. beam splitters, antireflection coatings, photonic mirrors)^27^. Solution processed chalcogenide glass multi-layered thin films are difficult to prepare in principle due to the fact that the subsequent layer etches the preceding layer during its deposition. Nevertheless, several approaches dealing with this issue have been described before^28,29^, none of them allows direct deposition of the next layer.

The majority of solution processed chalcogenide thin film research has been done on glasses from an As-S compositional system (mostly As_2_S_3_), which will hardly reach any practical application due to the toxicity of the arsenic and low chemical resistance of prepared films. Lately, we can observe a shift of the scientific interest to non-toxic Ge-based glasses^30,31^, particularly to glasses from Ge–Sb–S system^32–34^.

The presented manuscript provides characteristics of Ge_20_Sb_5_Se_75_ solution processed thin films, another non-toxic composition suitable for practical applications. The dissolution of Ge–Sb–Se chalcogenide glass compositional system in amine-based solutions^35,36^ has been previously reported. Nevertheless, compact thin film in specular quality necessary for application in optics have not been yet investigated. The optical properties of prepared thin films were studied in dependence on the annealing temperature in order to study the thermoinduced compositional and structural changes occurring in the thin film during annealing. Our previous work on Ge_20_Sb_5_S_75_ amorphous thin films revealed significant chemical resistance of hard-baked thin films allowing direct repeated depositions of the glass solution on the thin film to reproducibly prepare thicker samples^37^. The hard-baked Ge_20_Sb_5_Se_75_ thin films studied in the presented paper showed remarkably high chemical resistance as well, which in combination with high refractive index allowed their use for production multilayered photonic materials. Alternating depositions of Ge_20_Sb_5_S_75_ and Ge_20_Sb_5_Se_75_ films were used for the simple preparation of multi-layers systems with significantly different optical properties of the separate layers. Such approach could be potentially highly advantageous as it will allow to prepare the photonic elements using exclusively solution deposition techniques, well-developed on industrial production scale. Finally, the possibility of mixing the solutions of both glasses was verified in order to offer the way to precisely tailor the optical properties of each layer, which also significantly widens the available chemically resistant chalcogenide glass compositions suitable for multilayer stacking.

## Experimental details

The source bulk chalcogenide glasses of Ge_20_Sb_5_S_75_ and Ge_20_Sb_5_Se_75_ compositions were synthesized using the standard melt-quench method. Required amounts of pure (5N) elements were weighted into cleaned quartz ampoules and sealed under vacuum (10^−3^ Pa). The syntheses were conducted in a rocking tube furnace at 950 °C for 32 h. After synthesis, the ampoules were quenched in cold water.

The bulk glasses were crushed in an agate bowl and afterward dissolved in solvent consisting of 10 vol.% methanol (MeOH) and 90 vol.% n-propylamine (PrNH_2_) under a nitrogen atmosphere in a glovebox. The concentration of both solutions was 0.075 g of glass to 1 ml of solvent mixture. Glass dissolution was performed at room temperature with stirring using magnetic stirrer. Obtained glass solutions were without any precipitations or turbidity.

Chalcogenide glass thin films were deposited by the spin-coating method. Soda-lime microscopic slides (1 mm thick) were cut to a square shape (25 × 25 mm) and used as substrates. Substrates were cleaned prior to deposition in ultrasonic bath and aqua regia to remove organic residua and improve adhesion. The chalcogenide glass solutions were pipetted onto rotating soda-lime substrates and spin-coated (spin-coater Laurell WS-650Mz-23NPPB) for 60 s at a spin-rate of 4000 RPM for the Ge_20_Sb_5_S_75_ solutions and 2000 RPM for Ge_20_Sb_5_Se_75_ and mixed Ge_20_Sb_5_S_37.5_Se_37.5_ composition. Deposited thin films were annealed at 60 °C on a hot plate for 20 min (hereafter referred to as “as-prepared” thin films). The as-prepared thin films were subsequently annealed at temperatures 110, 160, and 210 °C for 1 h on a hot plate. The temperature range was chosen based on the preliminary experiments and our previous work with Ge_20_Sb_5_S_75_ glass^33^. Samples annealed at 210 °C are hereinafter referred to as “hard-baked”. All depositions and annealing of the thin films were performed in a glovebox with a pure nitrogen atmosphere (Unilab Pro SP 1800/780).

Double-layered thin films were prepared by spin-coating glass solution onto previously prepared thin films annealed at 210 °C (hard-baked). Prepared double-layers were subsequently annealed at 210 °C for 1 h on a hot plate in a nitrogen-filled glovebox. Triple-layered thin films were prepared from double-layered thin films using the same approach.

The optical properties of thin films and multilayers were determined using variable-angle spectroscopic ellipsometer (VASE, J.A. Woollam) with a rotating analyzer in the spectral range of 210–1700 nm. Measurements were carried out using 25 analyzer revolutions with photon energy steps of 0.05 eV at three selected angles of incidence (AOI, i.e., 50, 60, and 70°). The measured ellipsometry data were evaluated in WVASE32 software together with transmission spectra (measured using UV–VIS-NIR spectrometer UV-3600, Shimadzu) as supporting material for generated data fit. Minimization procedure using the mean square error (MSE, for its definition see e.g., Eq. 5 in^38^) values between measured and generated data was conducted to calculate geometrical parameters and refractive index of deposited thin films and multilayers. Their surface roughness was modeled using Bruggerman type of effective medium approximation of void and layers^39^.

Surface morphology of studied single- and multi-layered thin films was investigated using atomic force microscopy (AFM) in semi-contact mode using NTEGRA microscope (NT-MDT) equipped with NSG 10 (AppNano). Three areas of 5 × 5 µm were measured for each hard-baked thin film. The presented values of RMS (calculated according to ISO 4287/1 norm) are the average values of three scans with error bars representing standard deviations.

The structure of thin films was studied using Raman spectroscopy. Measurements were performed on MultiRAM (Bruker) FT-Raman spectrometer using 1064 nm Nd:YAG excitation laser (resolution 2 cm^−1^, average of 64 scans). The resulting spectra of thin films were normalized by the intensity of the most intensive band.

A LYRA 3 scanning electron microscope (Tescan) equipped with an EDS analyzer Aztec X-Max 20 (Oxford Instrument) was used for both SEM scan measurement and a study of elemental composition. For the study of elemental composition, samples were measured on five 400 × 400 µm areas at accelerating voltage of 5 kV. The presented data show the average of five measurements, and the standard deviation is given as an error bar. The SEM scans were acquired at 10 kV acceleration voltage.

The chemical resistance of the thin films was studied by etching thin films in a solution of 2 vol.% ethylenediamine (EDA) in dimethyl sulfoxide (DMSO) at 25 °C. The average etching rates were evaluated as described in^40^.

## Results and discussion

**Figure 1:**
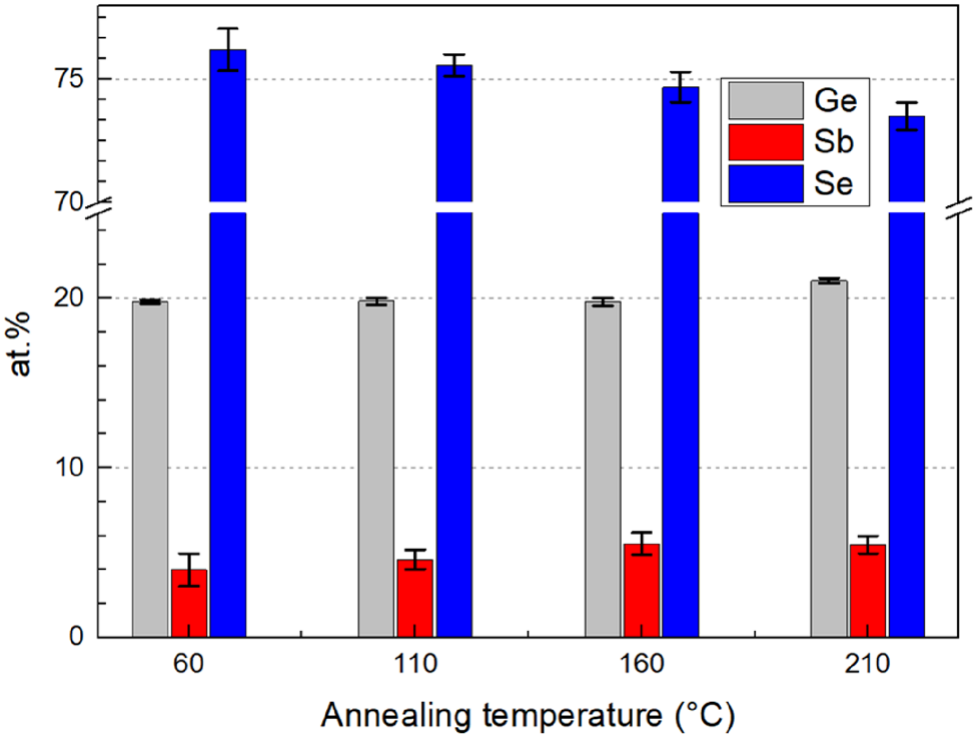
Annealing temperature dependence of the glass composition.

The composition of as-prepared and annealed Ge_20_Sb_5_Se_75_ thin films was studied using EDS (Fig. 1). Obtained data show minor depletion of Se with increasing annealing temperature. A probable cause could be expected in partial Se evaporation due to its significant overstoichiometry in the glass. The observed phenomenon is in good agreement with similar results described on solution processed glasses from Ge-Se compositional system^30^.

**Figure 2:**
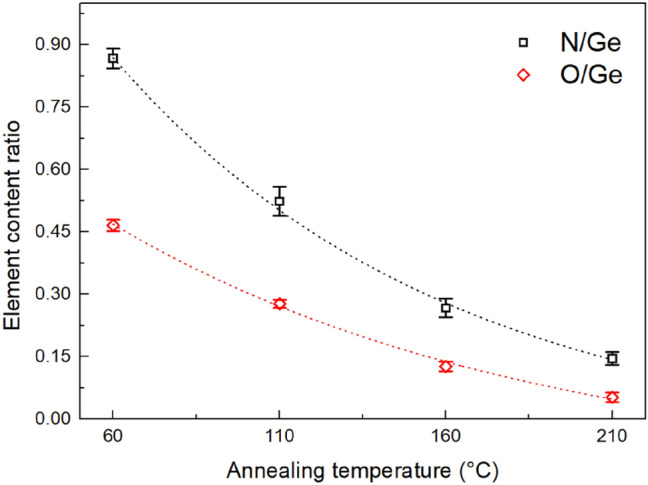
Annealing temperature dependence of the N/Ge and O/Ge elemental ratios in the thin films. Dotted lines are provided only to guide your eyes.

Besides the elemental composition of the glass, the contents of nitrogen and oxygen in the thin films were also studied in dependence on the annealing temperature (Fig. 2). The content of the nitrogen in the thin film is directly linked to the content of the organic residua of PrNH_2_ incorporated in the structure of the glass^30,41^. Because all thin films were deposited and also annealed in oxygen free atmosphere, content of the oxygen in the thin films is mainly connected with residua of MeOH, which was used in the solvent mixture. Figure 2 gives evidence that both nitrogen and oxygen contents decrease significantly with increasing annealing temperature, which proves release of the organic residua from the structure of the thin film. It is worth mentioning that hard-baked thin films show significantly lower content of nitrogen in comparison to sulfur-based thin films of analogous composition Ge_20_Sb_5_S_75_^37^.

**Figure 3:**
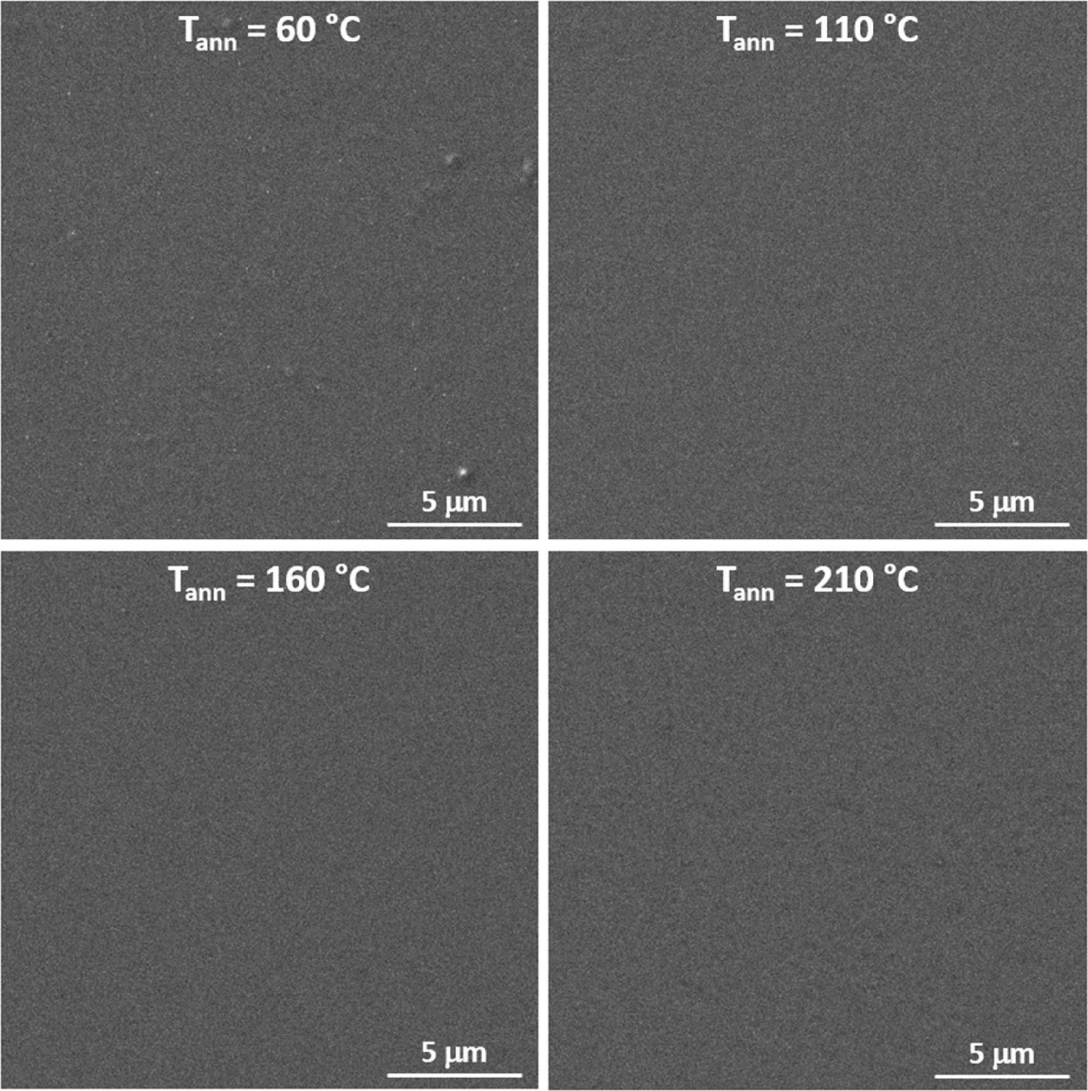
SEM scans of Ge_20_Sb_5_Se_75_ thin films annealed at studied temperatures.

The surface quality of the thin films was investigated using SEM. Figure 3 shows smooth surface of all studied thin films without the presence of any structures, crystals, or cracks. Additionally, values of surface roughness well below 1 nm (obtained from optical ellipsometry—Table 1) verify the optical quality of the thin films’ surfaces at all annealing temperatures. Surface roughness remains low despite significant shrinkage in the thin films thicknesses (up to ~ 1/3 of the thin film’s original thickness), which in the case of solution processed thin films often leads to an increase in surface roughness^37,42^. The decrease of thin films thickness is caused by release of organic residua from the thin films and structural polymerization, which will be discussed later. Transmission spectra of Ge_20_Sb_5_Se_75_ thin films annealed at studied temperatures proving the good optical quality of the samples, are provided in supplemental materials in Fig. S1.

**Table 1:** Values of surface roughness, thickness and thickness shrinkage of Ge_20_Sb_5_Se_75_ thin films annealed at studied temperatures.

T_ann_ (°C)	Surf. roughness (nm)	Thickness (nm)	Thickness shrinkage
60	0.8 ± 0.2	251 ± 5	0%
110	0.8 ± 0.2	236 ± 5	6%
160	0.7 ± 0.2	182 ± 5	27%
210	0.6 ± 0.2	167 ± 8	33%

**Figure 4:**
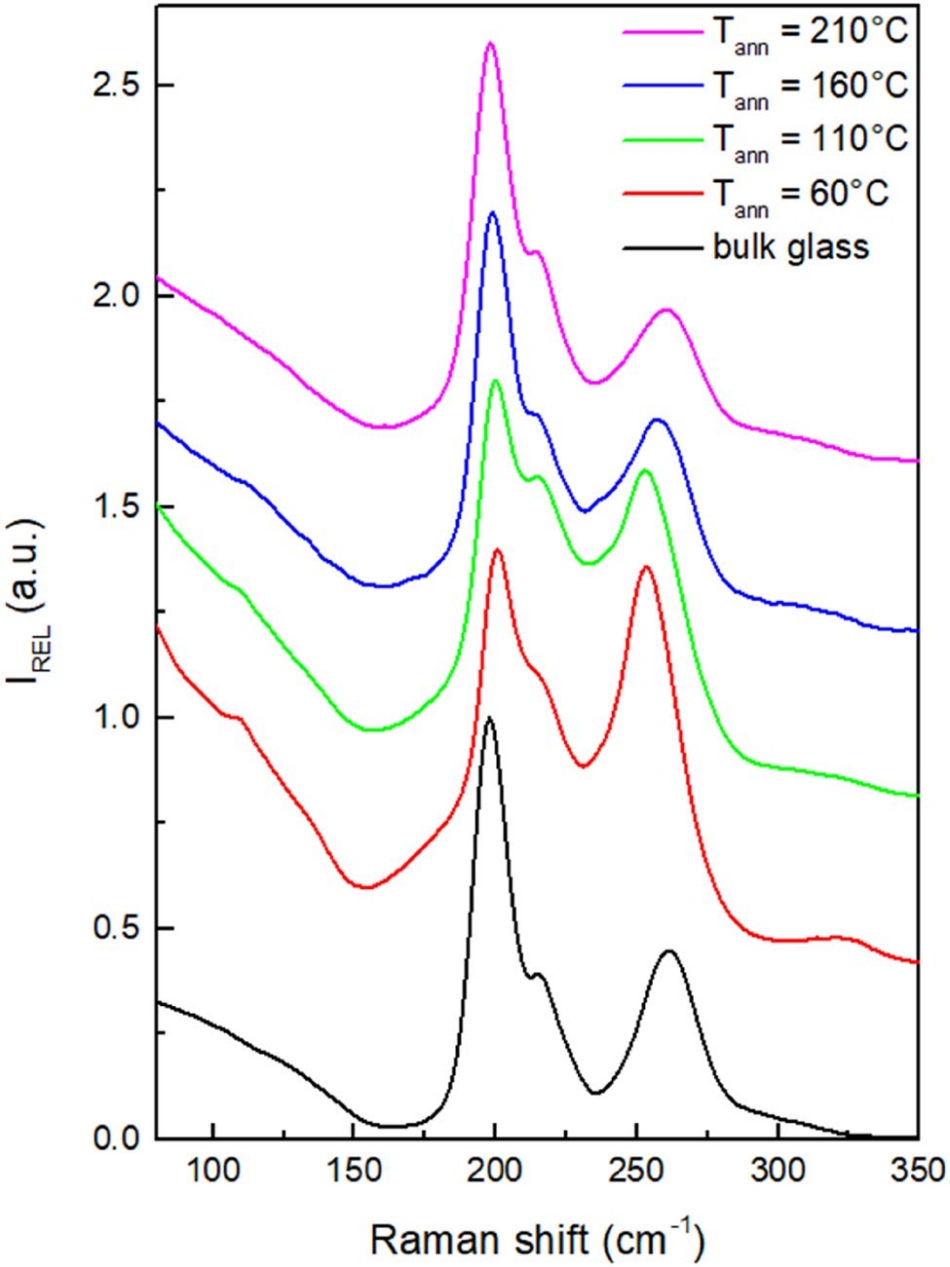
Raman spectra of source bulk Ge_20_Sb_5_Se_75_ glass and thin films annealed at studied temperatures.

The structure of the source Ge_20_Sb_5_Se_75_ bulk glass, as well as of all studied thin films, was investigated using Raman spectroscopy (Fig. 4). Structure of the bulk glass consists predominantly of corner-shared GeSe_4/2_ tetrahedrons providing the most intensive band at 199 cm^−1^ in Raman spectra^43–45^. Additionally, band at 216 cm^−1^ corresponds to the presence of edge-shared GeSe_4/2_ tetrahedrons^43–45^. The second most intensive band in Raman spectra at 262 cm^−1^ represents the Se–Se vibrations in Se chains (259 cm^−1^) and Se_n_ rings (268 cm^−1^)^46^. Band related to the vibration Sb–Se bond should be situated at ~ 190 cm^−1^^46^, but it is not visible due to the low content of Sb in the glass and near presence of the most intensive band in the spectra.

The structure of as-prepared solution processed thin films differs significantly from the structure of the source bulk glass. The most intensive band in Raman spectra remains the band at 199 cm^−1^ related to the vibration of corner-shared GeSe_4/2_ tetrahedrons. The intensity of the band at 258 cm^−1^ increases significantly, which suggests the separation of Se chains and/or rings from the structure of the glass. Correspondingly, the intensity of the band at 216 cm^−1^ (edge-shared GeSe_4/2_ tetrahedrons) increases, and shoulder on the main band at 175 cm^−1^ appears. The presence of this shoulder can be attributed to Ge–Ge vibration in Ge_2_Se_6/2_ structural unit^46,47^. An increase in the intensity of the background between the two main bands and the presence of a new band at 110 cm^−1^ suggests the presence of the amorphous Se^30,48^. Another new band at 323 cm^−1^ occurs in the Raman spectrum of as-prepared thin film. This band was completely missing in the spectrum of source bulk glass. It has been attributed to the vibration of organic–inorganic salts^30^—products of dissolution of the source bulk glass in the solvent mixture 10% MeOH in PrNH_2_.

It is worth mentioning that even though no bands corresponding to Sb species are apparent in the Raman spectrum of as-prepared thin film, Sb presence in the thin film diminishes the separation of Se during the glass dissolution. Slang et al.^30^ studied Ge_25_Se_75_ solution processed thin films (same content of Se) deposited from the same solvent composition and stabilized in the same manner. The Ge_25_Se_75_ Raman spectra showed significantly higher concentration of Se separated from the glass structure than we observed in the case of Ge_20_Sb_5_Se_75_ thin films.

Annealing of the as-prepared thin films induces changes in the thin films, which incrementally shift the structure toward the structure of the source bulk glass. Se atoms from chains and rings are being incorporated into the polymer structure of the glass, decreasing the content of the edge-shared tetrahedrons and Ge_2_Se_6/2_ structural units. Dissolution products (organic–inorganic salts) are decomposed, which is in good agreement with the release of organic residua observed in EDS analyses.

**Figure 5:**
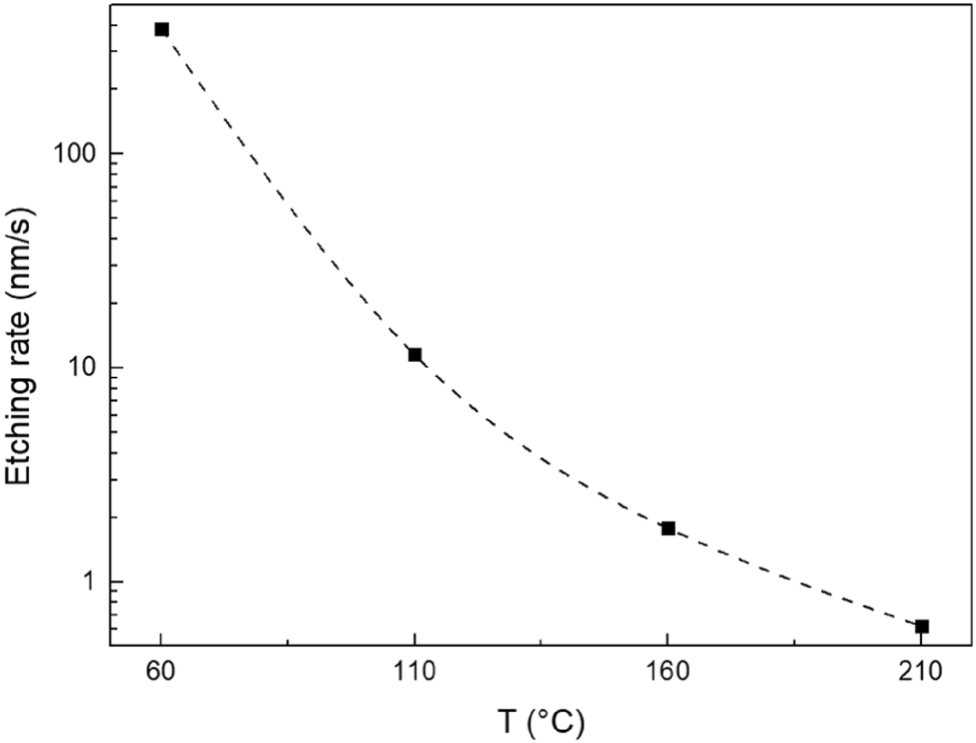
Annealing temperature dependence of Ge_20_Sb_5_Se_75_ thin films average etching rates. Dashed line is provided only to guide your eyes.

The chemical resistance of studied Ge_20_Sb_5_Se_75_ thin films was investigated by measuring etching kinetics in 2% EDA/DMSO solution. Average etching rates of thin films in dependence on the annealing temperatures are provided in Fig. 5. Significant drop of etching rate with increasing annealing temperature is in good agreement with the etching behaviour of solution processed thin film of many compositions^33,42,49^. The cause of this phenomenon can be found in the release of organic residua and structural polymerization during the annealing of the thin films as discussed above in the paragraph regarding Raman spectroscopy.

Optical parameters (refractive index and extinction coefficient) and thickness (partially discussed above) of studied thin films were measured using optical ellipsometry. Spectral dependence of optical constants was modelled using the Tauc-Lorentz oscillator^50,51^ typical for amorphous chalcogenides^31,33^. In addition to our previous study focused on spin-coated Ge_20_Sb_5_S_75_ thin films^33^, the possibility of refractive index gradient within the thin film was tested. The model including a sub-layer with a higher refractive index on the top of the thin film and a sub-layer with a lower refractive index at the bottom of the thin film was found to significantly decrease mean square error (MSE, i. e. significantly increase the goodness of the fit), which is consistent with the results published on thicker chalcogenide films^52^. An example of measured ellipsometry data together with the best fits with/without bottom and top sub-layers is provided in the supplementary materials Fig. S2. Therefore, a structure with bottom and top sub-layers with a different refractive index will be used for the description of each layer of spin-coated thin films in the following.

In order to decrease the number of fit parameters, the parameters *A*, *E*_0_, *C* and *E*_g_ (standing for amplitude, peak position, broadening, and energy onset of the oscillator—also energy bandgap of the material) describing imaginary part of dielectric function were kept constant for the bottom as well as the top sub-layer. Furthermore, one additional parameter describing the increase of the real part of dielectric function for top sub-layer with respect to bottom sub-layer was added. Totally four layers, including glass substrate with optical constants measured separately, bottom sub-layer, top sub-layer, and surface roughness, will be used to model the inner structure of the spin-coated thin film. This approach will be used consistently for all studied single-layered thin films.

**Figure 6:**
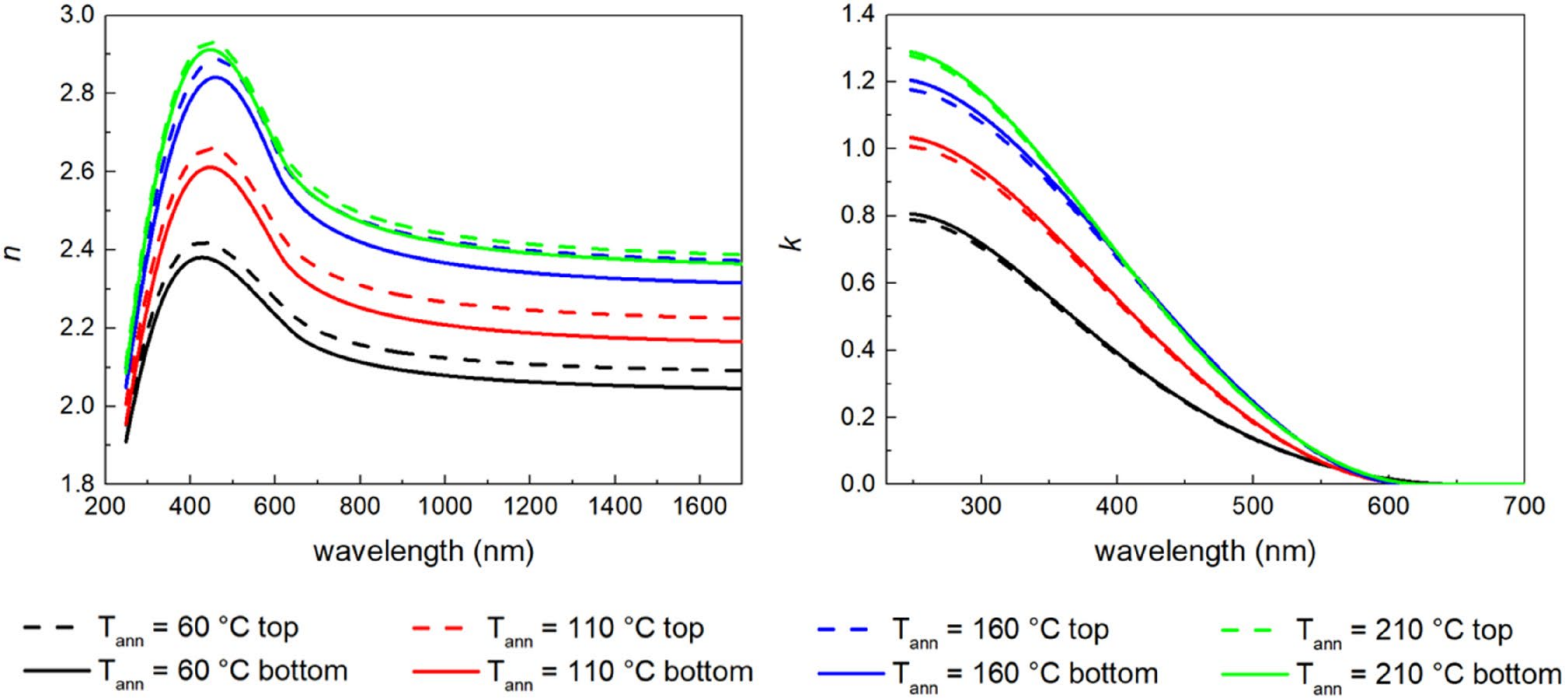
Spectral dependences of refractive index (left) and extinction coefficient (right) for Ge_20_Sb_5_Se_75_ thin films annealed at studied temperatures (considering gradient of optical parameters in the thin film modelled as bottom and top sub-layer).

Spectral dependences of the optical constants for Ge_20_Sb_5_Se_75_ thin films annealed at various temperatures are depicted in Fig. 6 (refractive index—left, extinction coefficient—right). The expected error is ~ 0.01 for both the refractive index as well as extinction coefficient. The increase of the refractive index, together with the steeper increase of the extinction coefficient with the increasing annealing temperature can be attributed to the release of organic residua (as was discussed in EDS analyses section) and polymerization of the structure (as was discussed in Raman spectroscopy section). Observed phenomena are consistent with previous studies of solution processed amorphous chalcogenide materials^31,33,53,54^. Figure 6 shows that the refractive index of the top sub-layer is slightly higher for all annealing temperatures (~ 0.05 for annealing temperatures 60–160 °C and ~ 0.02 for annealing temperature 210 °C for wavelength 1550 nm). The cause can be found in the depletion of the thin films surface (top sub-layer) from the organic residua due to the shorter diffusion path to the surface, which is in good agreement with results published in^52^ observed on thin films from As–S compositional system. Furthermore, smaller differences in refractive index between top and bottom sub-layer can be found in the hard-baked thin films annealed at 210 °C. Annealing at the higher temperature would loosen the glass structure more, thus easing the release of the organic residua from the lower part of the thin film (bottom sub-layer) resulting in an increase of the refractive index.

**Scheme 1:**
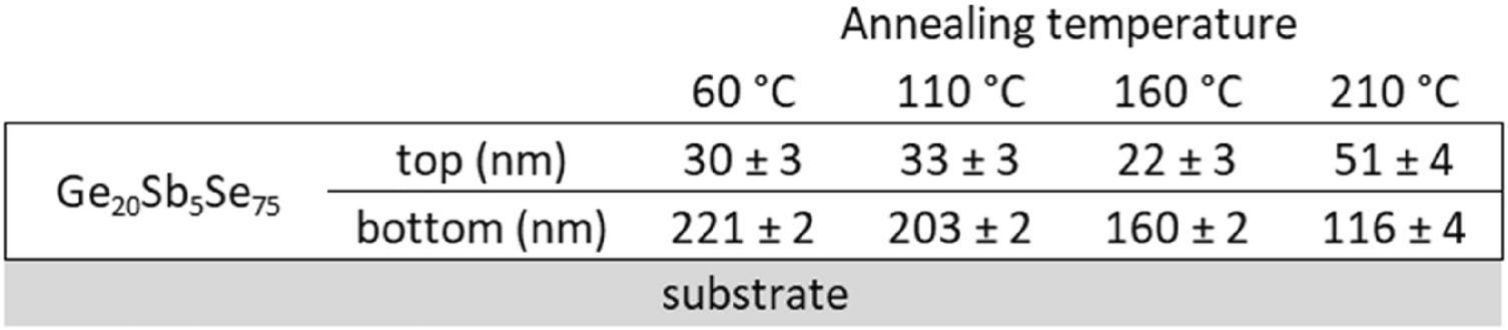
Values of the thickness of the bottom and top sub-layers of Ge_20_Sb_5_Se_75_ thin films annealed at studied temperatures.

Thicknesses for the bottom and top sub-layers obtained by the best fit of the ellipsometry data for all annealing temperatures are summarized in Scheme 1. The determination accuracy of the thicknesses for the bottom and top sub-layer depends on the refractive index contrast between them and therefore is lower in the case of hard-baked film where the increase for the refractive index of the top sub-layer compared to the bottom sub-layer is the lowest.

For further experiments, thin films of Ge_20_Sb_5_S_75_ were prepared and analyzed in the same manner as the Ge_20_Sb_5_Se_75_ thin film (i.e. using the same solvent mixture MeOH-PrNH_2_, hard-bake temperature 210 °C and evaluation of optical properties as a double layered thin film with bottom and top sub-layer). Our previous works^21,22^ demonstrated the possibility of altering (tailoring) of the thin films optical properties by mixing solutions of two different chalcogenide glasses with different optical properties. Thin films prepared from mixed solutions then showed linear combinations of optical properties of the two source glasses. The same process can be used even in the case of Ge_20_Sb_5_Se_75_ and Ge_20_Sb_5_S_75_ glasses to tailor the optical properties of the thin film. For demonstration, thin films of mixed composition Ge_20_Sb_5_S_37.5_Se_37.5_ were prepared using a mixture of Ge_20_Sb_5_Se_75_ and Ge_20_Sb_5_S_75_ source solutions. Obtained thin films of quaternary composition are hereinafter referred to as “mixed composition”.

**Scheme 2:**
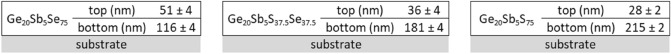
Values of the thickness of the bottom and top sub-layers of Ge20Sb5Se75, mixed composition Ge20Sb5S37.5Se37.5 and Ge20Sb5S75 thin film hard-baked at 210 °C.

**Figure 7:**
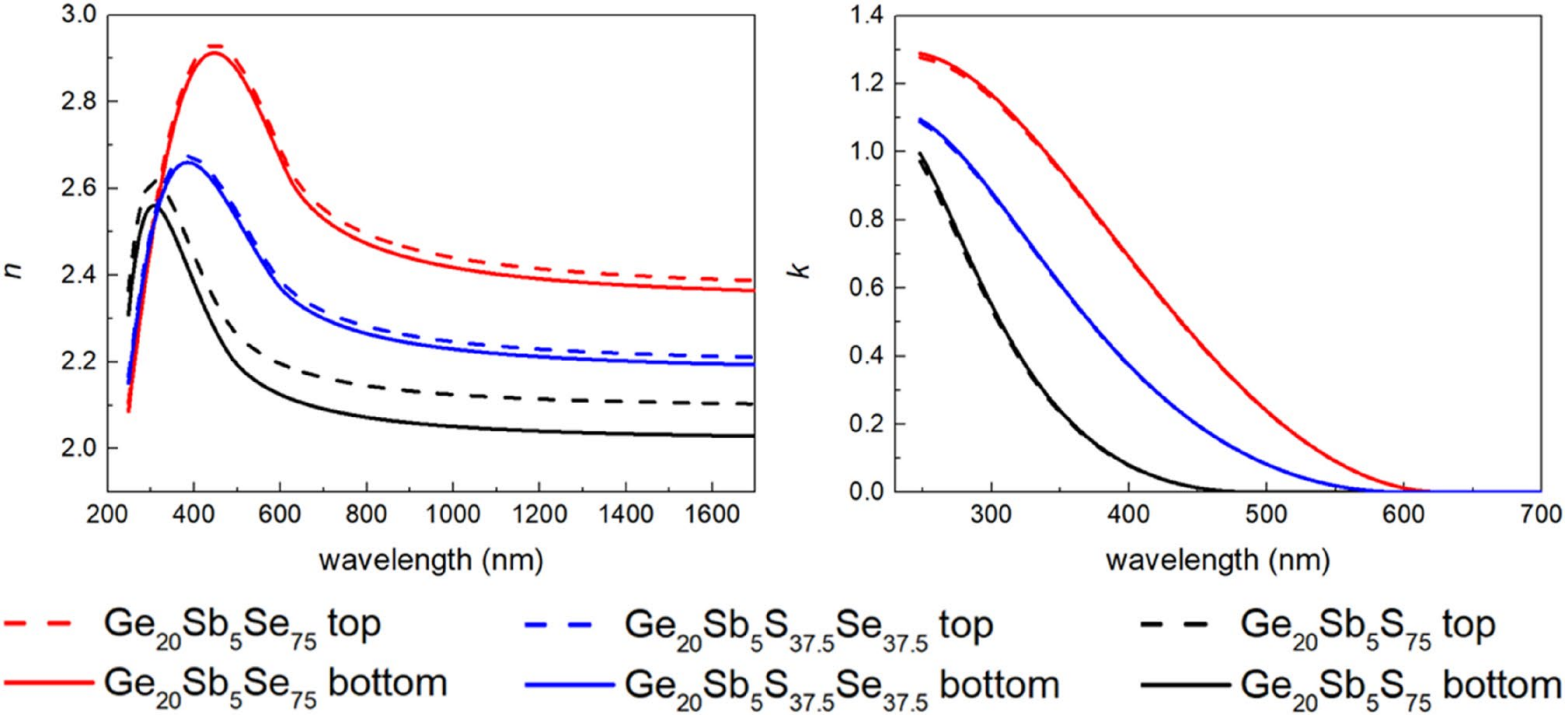
Spectral dependence of the refractive index (left) and extinction coefficient (right) for hard-baked Ge_20_Sb_5_Se_75_, Ge_20_Sb_5_S_37.5_Se_37.5_ (mixed composition) and Ge_20_Sb_5_S_75_ thin films (considering gradient of the refractive index films modelled by bottom and top sub-layer).

Optical analysis considering the top and bottom sub-layer of the film was conducted for hard-baked spin-coated Ge_20_Sb_5_S_75_ films and hard-baked spin-coated film of mixed composition (Ge_20_Sb_5_S_37.5_Se_37.5_). Thicknesses of the top and bottom sub-layer are summarized in Scheme 2. Note that total layer thickness for hard-baked layers is decreasing from ~ 240 nm for Ge_20_Sb_5_S_75_ to ~ 170 nm for Ge_20_Sb_5_Se_75_ with ~ 220 nm for mixed composition Ge_20_Sb_5_S_37.5_Se_37.5_. Also, a slight increase in the thickness of the top sub-layer can be noticed when replacing S with Se in the layer composition. A comparison of the spectral dependence of the optical constants for hard-baked thin films of all compositions is given in Fig. 7. Expected error is ~ 0.01 for both the refractive index as well as the extinction coefficient. One can notice an increase of the refractive index connected with the red shift of the energy bandgap when replacing S with Se (energy bandgap for Ge_20_Sb_5_S_75_ is ~ 2.5 eV, for Ge_20_Sb_5_Se_75_ ~ 2.0 eV and for Ge_20_Sb_5_S_37.5_Se_37.5_ ~ 2.1 eV). Also, the difference between the values of the refractive index for the bottom and top sub-layer decreases when S is replaced by Se (~ 0.07 for Ge_20_Sb_5_S_75_, ~ 0.03 for mixed composition, and ~ 0.02 for Ge_20_Sb_5_Se_75_ at the wavelength 1550 nm) which relates to the increase of the top sub-layer thickness and to lower accuracy of the presented thickness values.

**Scheme 3:**
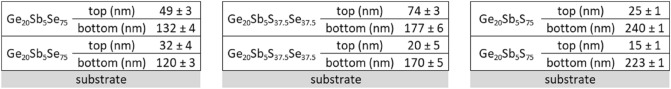
Values of thickness of the bottom and top sub-layers for hard-baked double layered Ge_20_Sb_5_Se_75_, mixed composition Ge_20_Sb_5_S_37.5_Se_37.5_ and Ge_20_Sb_5_S_75_ thin films.

Significant chemical resistance of hard-baked Ge_20_Sb_5_Se_75_ thin films opens the way for repeated depositions (stacking of layers) in order to increase the thickness of the thin film. The same process was used in our previous publication studying solution processed thin film of Ge_20_Sb_5_S_75_ chalcogenide glass deposited from n-butylamine-based solution. Double-layered thin films of Ge_20_Sb_5_Se_75_ were successfully prepared in specular optical quality as well as thin films of mixed composition Ge_20_Sb_5_S_37.5_Se_37.5_. Consistently with evaluation methodology, each layer is modelled using bottom and top sub-layer. Optical constants for double-layered thin films evaluation were taken from the analysis of single-layered thin film without any change. Corresponding thicknesses obtained from the best fit of the ellipsometry data measured for a double-layered thin film of Ge_20_Sb_5_Se_75_, mixed composition Ge_20_Sb_5_S_37.5_Se_37.5_ and Ge_20_Sb_5_S_75_ are summarized in Scheme 3 where the position of the glass substrate is also indicated.

It is worth mentioning that the total thickness of the first layer (on substrate) in the double-layered structure is slightly lower in comparison to previous results (see Scheme 1) and also in comparison with the total thickness of the second layer for all measured compositions (Ge_20_Sb_5_Se_75_, Ge_20_Sb_5_S_75_ as well as mixed Ge_20_Sb_5_S_37.5_Se_37.5_). The cause can be probably found in etching of the preceding film, during the process of the spin-coating of the second layer. On the other hand, obtained thicknesses are very close to the results from single layers (see Scheme 1), confirming the possibility of layer stacking. Moreover, surface roughness obtained from ellipsometry data analysis is below 2 nm indicating good optical quality even for double-layered structure.

Significant chemical resistance of both Ge_20_Sb_5_Se_75_ and Ge_20_Sb_5_S_75_ hard-baked thin films allows the preparation of multi-layered thin film with alternating S-based and Se-based chalcogenide glasses by simple deposition of the next layer by spin-coating on the hard-baked previous layer without the need for any additional treatment or deposition of any separating layer. Double-layered Ge_20_Sb_5_Se_75_/Ge_20_Sb_5_S_75_ and Ge_20_Sb_5_S_75_/Ge_20_Sb_5_Se_75_ thin films were prepared using mentioned approach. Consistently with previous evaluation of optical constants each layer is modelled using bottom and top sub-layers.

**Scheme 4:**
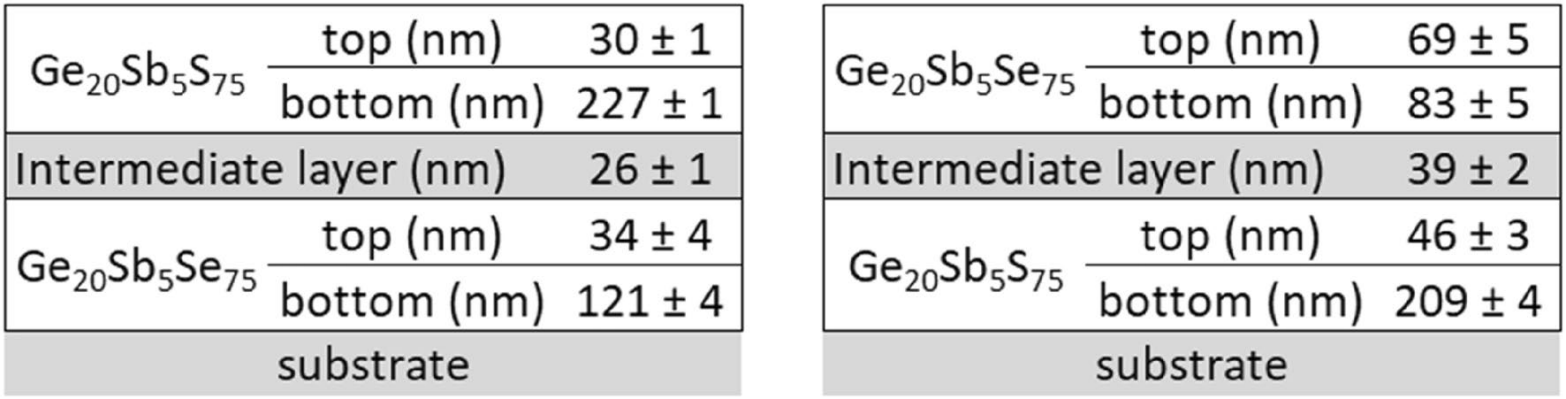
Values of thicknesses of the bottom and top sub-layers as well as of intermediate layer for hard-baked double layered Ge_20_Sb_5_Se_75_/Ge_20_Sb_5_S_75_ and Ge_20_Sb_5_S_75_/Ge_20_Sb_5_Se_75_ thin films.

Adopting 5 layer model structure (respectively 6 layers when surface roughness is also considered in the model) similar to that presented in Scheme 3 is leading to relatively high values of MSE (~ 11 in both cases). Quality of the fit can be significantly improved, i.e. MSE significantly decreases, when then intermediate layer is introduced between Ge_20_Sb_5_S_75_ and Ge_20_Sb_5_Se_75_ layers (thus 7 layers in total in the model). Optical properties of this intermediate layer are modelled using Bruggerman type of effective medium approximation of both layers (more specifically using 50% of top sub-layer under and 50% of bottom sub-layer above the intermediate layer). Adding one more parameter (thickness of the layer) decreases value of MSE to ~ 4 for Ge_20_Sb_5_Se_75_/Ge_20_Sb_5_S_75_, which in our opinion strongly suggests the existence of the intermediate layer. An example of measured ellipsometry data together with the best fits with/without intermediate layer are provided in the supplementary materials Fig. S3. Corresponding thicknesses obtained from the best fit of the ellipsometry data for double layered thin films (Ge_20_Sb_5_Se_75_/Ge_20_Sb_5_S_75_ and Ge_20_Sb_5_S_75_/Ge_20_Sb_5_Se_75_) are summarized in Scheme 4. Surface roughness obtained from ellipsometry data analysis is below 3 nm.

The presence of the intermediate layers could have two potential origins. Firstly, the underlying thin film can be partially etched (dissolved) during the deposition of the following layer and etching products mixed with the solution of the next layer. Even though hard-baked thin films are chemically very resilient, the solution of the glass is often more chemically aggressive than the solvent mixture itself. For instance, recently published paper showed that amorphous Se dissolves in As_40_S_60_ glass solution, although solvent alone doesn’t dissolve Se at all^16^. The second possible reason for the formation of the intermediate layer could be the diffusion of S and Se into the opposite layers during the hard-bake process, which follows after each deposition cycle.

**Scheme 5:**
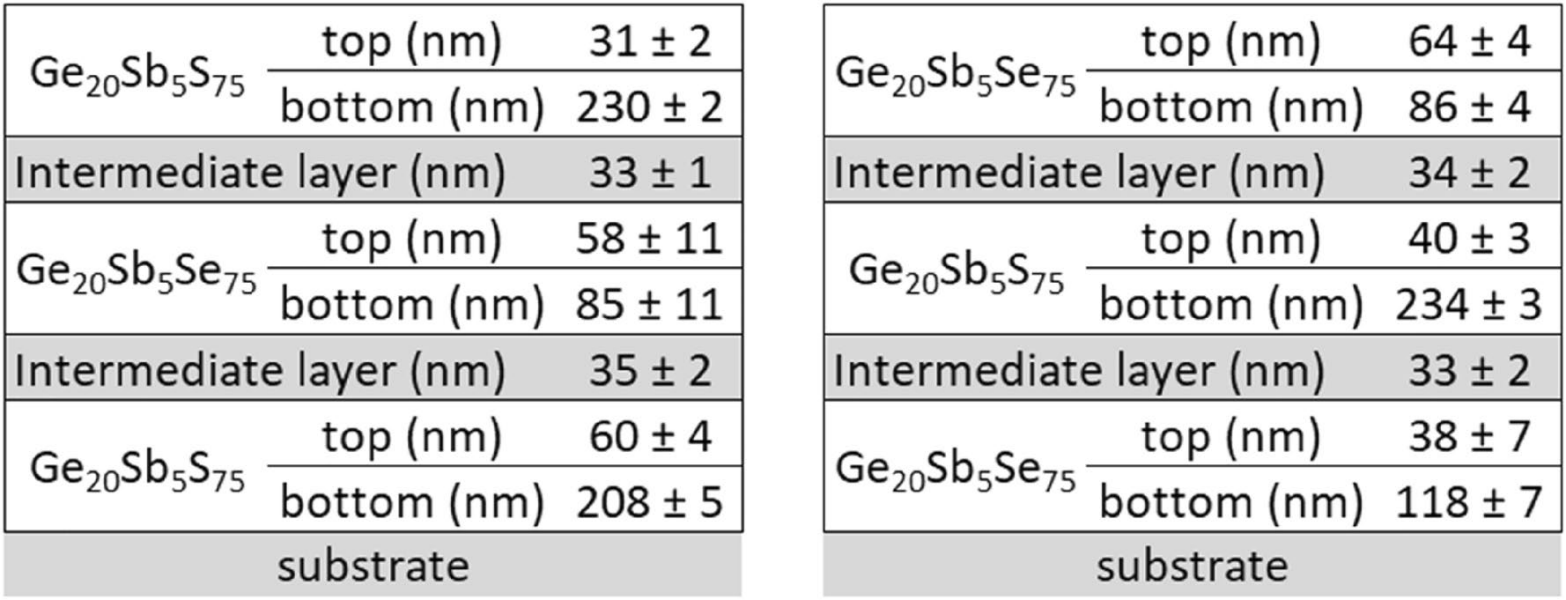
Values of the thickness of the bottom and top sub-layers for hard-baked triple-layers of Ge_20_Sb_5_S_75_/Ge_20_Sb_5_Se_75_/Ge_20_Sb_5_S_75_ and Ge_20_Sb_5_Se_75_/Ge_20_Sb_5_S_75_/Ge_20_Sb_5_Se_75_.

Finally, triple-layered thin films of Ge_20_Sb_5_S_75_/Ge_20_Sb_5_Se_75_/Ge_20_Sb_5_S_75_ and Ge_20_Sb_5_Se_75_/Ge_20_Sb_5_S_75_/Ge_20_Sb_5_Se_75_ glasses were prepared. Following previously established approach, each layer is presented by bottom a top sub-layers and an intermediate layer is added between the layers of different compositions for evaluation of the layers thicknesses by optical ellipsometry. Therefore 9 layers (respectively 10 layers when the surface roughness is also considered in the model) are used in order to model the inner structure of a triple-layered thin film. Unchanged optical constants of these layers described previously were also used in this case. Corresponding thicknesses obtained from the best fit of the ellipsometry data are summarized in Scheme 5. Even for the triple-layered structure, the values of the surface roughness obtained from ellipsometry data analysis are below 3 nm.

Overall, even for triple-layered thin films, the thicknesses of the layers correspond to that discussed previously for simpler structures (single and double layers). Significantly lower values of MSE are achieved by adding intermediate layers, confirming the existence of intermediate layers.

Large area SEM scans (scan size 1500 × 1500 µm) together with micrometer-scaled AFM scans (scan size 5 × 5 µm) presented in supplementary material (Figures S4 and S5) demonstrate the high surface quality of both single-layered thin films and multi-layered thin films, with no sign of cracks, distortion or other microscopic defects.

**Figure 8:**
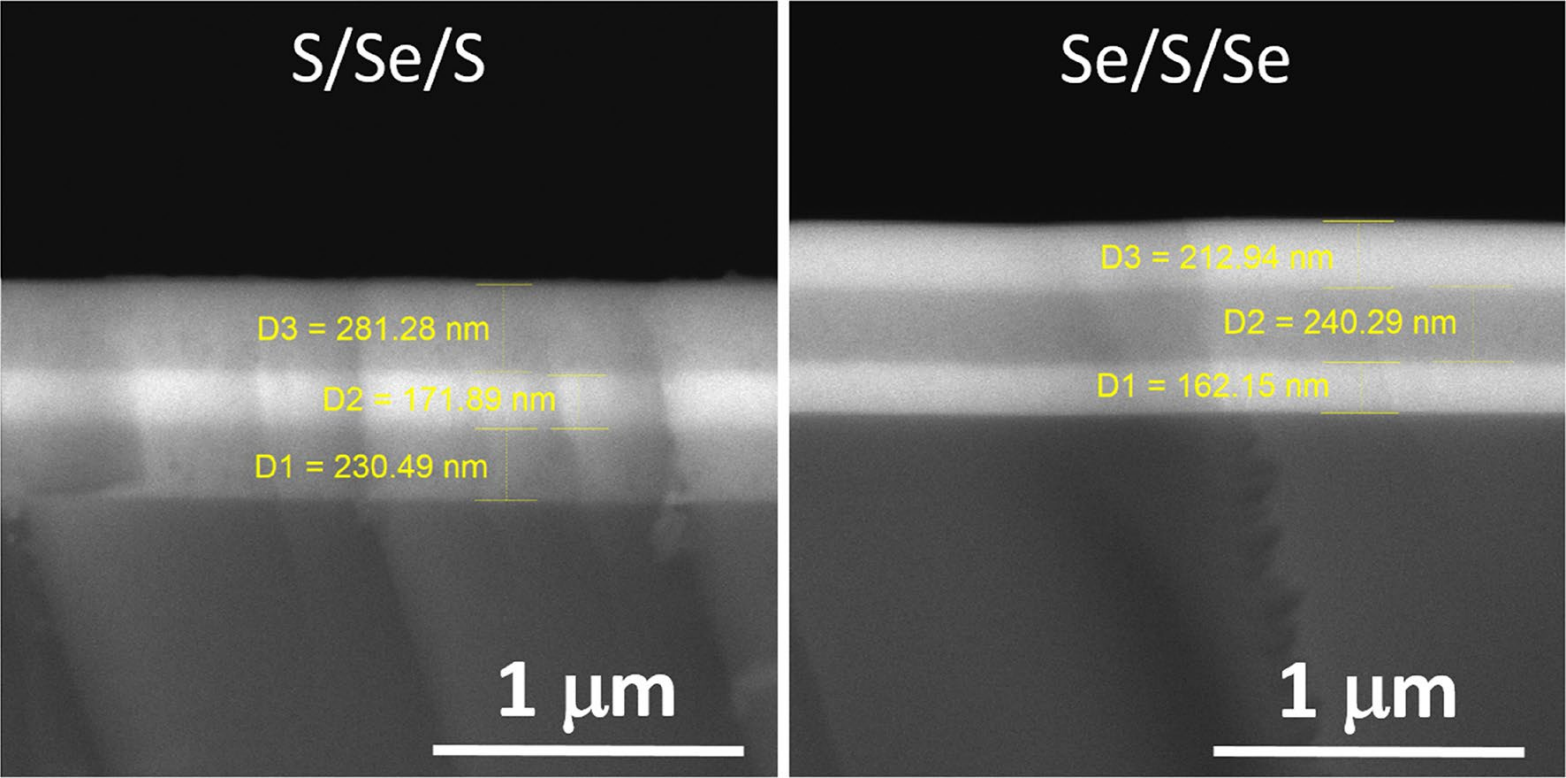
SEM scans (back-scattered electrons) of multi-layered thin films Ge_20_Sb_5_S_75_/Ge_20_Sb_5_Se_75_/Ge_20_Sb_5_S_75_ (left) and Ge_20_Sb_5_Se_75_/Ge_20_Sb_5_S_75_/Ge_20_Sb_5_Se_75_ (right). Simplified labelling in the picture is used (S stands for Ge_20_Sb_5_S_75_ layers and Se for Ge_20_Sb_5_Se_75_ layers).

Low values of surface roughness obtained by spectroscopic ellipsometry for single and multi-layered thin films were verified using AFM. Surface roughness values obtained by evaluation of the AFM scans according to ISO 4287/1 norm are presented in supplementary materials in Table 1S. SEM scans acquired in back-scattered electrons of both studied Ge_20_Sb_5_S_75_/Ge_20_Sb_5_Se_75_/Ge_20_Sb_5_S_75_ and Ge_20_Sb_5_Se_75_/Ge_20_Sb_5_S_75_/Ge_20_Sb_5_Se_75_ multi-layered thin films are provided in Fig. 8. Scans clearly show the multi-layered structure of thin films. The diffusion character of the interfaces between chalcogenide layers verifies the presence of the intermediate layers (compare with interface with the substrate).

## Conclusion

Chalcogenide glass thin films of Ge_20_Sb_5_Se_75_ composition were successfully prepared from the glass solution in a solvent mixture of 10% methanol in n-propylamine by spin-coating method. The good optical quality of prepared thin films was achieved and maintained even after the hard-bake at 210 °C. Thermoinduced structural polymerization studied by Raman spectroscopy and release of organic residua observed by EDS resulted in a significant increase of refractive index and chemical resistance of the thin films. Optical ellipsometry showed the presence of gradient of optical parameters in the thin film, modelled as two sub-layers in studied thin films, from which the top sub-layer possesses a slightly higher refractive index. A probable cause of this phenomenon can be found in the fact, that sub-layer closer to the surface of the thin film contains less organic residua due to a shorter diffusion path to be released.

Significant chemical resistance of hard-baked Ge_20_Sb_5_Se_75_ thin films allowed repeated direct depositions of thin films by spin-coating, which enabled preparation of thicker thin films. Moreover, alternating depositions of previously studied Ge_20_Sb_5_S_75_ with Ge_20_Sb_5_Se_75_ thin films were used to prepare samples with the layered structure of materials with significantly different optical properties. Ellipsometry analysis showed the existence of the intermediate layer between the layers of different compositions.

Thin films of mixed composition Ge_20_Sb_5_S_37.5_Se_37.5_ were deposited from the source solution prepared by simple mixing of Ge_20_Sb_5_Se_75_ and Ge_20_Sb_5_S_75_ glass solutions. This process demonstrated the possibility of tailoring the optical properties of each separate layer in multi-layered samples according to the desired application.

Detailed investigation of non-toxic Ge_20_Sb_5_Se_75_ chalcogenide glass thin films prepared in specular optical quality from amine based solution significantly widens the possible applications of solution processed chalcogenide thin films. Simple preparation of multi-layered thin films of materials with different optical properties opens the door for the fabrication of advanced optical elements (e.g. beam splitters, photonic mirrors) via solution way.

### Supplementary Information

Supplementary Information.

## Data Availability

The gathered data are not publicly available but are available on reasonable request from the corresponding author.
