# Supplementary material for: Solution processed multi-layered thin films of Ge20Sb5S75 and Ge20Sb5Se75 chalcogenide glasses
Source: Sci Rep. 2023 Oct 3;13:16609. doi: 10.1038/s41598-023-43772-w (PMC10547712; doi:10.1038/s41598-023-43772-w)
Supplement: Supplementary file 1 — Supplementary Information. [file 41598_2023_43772_MOESM1_ESM.pdf]

Solution processed multi-layered thin films of $Ge_{20}Sb_5S_{75}$ and $Ge_{20}Sb_5Se_{75}$ chalcogenide glasses

Jiri Jemelka[1], Karel Palka[1,2*], Petr Janicek[2,3], Stanislav Slang[2], Jiri Jancalek[2], Michal Kurka[2], Miroslav Vlcek[1,2]

[1]Department of General and Inorganic Chemistry, Faculty of Chemical Technology, University of Pardubice, Studentska 95, 53210 Pardubice, Czech Republic

[2]Center of Materials and Nanotechnologies, Faculty of Chemical Technology, University of Pardubice, Studentska 95, 53210 Pardubice, Czech Republic

[3]Institute of Applied Physics and Mathematics, Faculty of Chemical Technology, University of Pardubice, Studentska 95, 53210 Pardubice, Czech Republic

*karel.palka@upce.cz

Supplemental materials

Fig. S1 Transmission spectra of substrate and $Ge_{20}Sb_5Se_{75}$thin films annealed at studied temperatures.

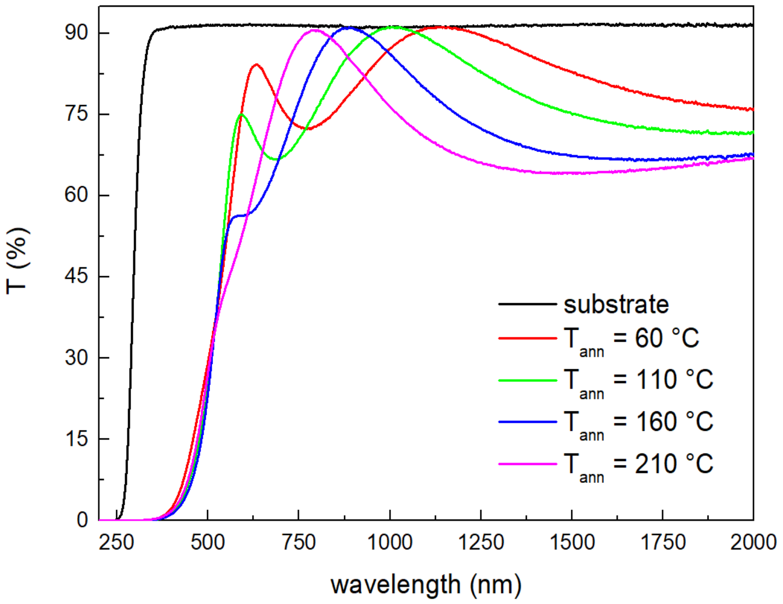

Fig. S2 Example of spectral dependence of ellipsometry parameter psi together two fits for angle of incidence 60 deg for hard baked single layer $Ge_{20}Sb_5S_{75}$. Squares are measured data, blue dashed line is the best fit using single layer model (MSE ~ 7) and full red line is the best fit using model with bottom and top sub-layer (MSE ~ 4).

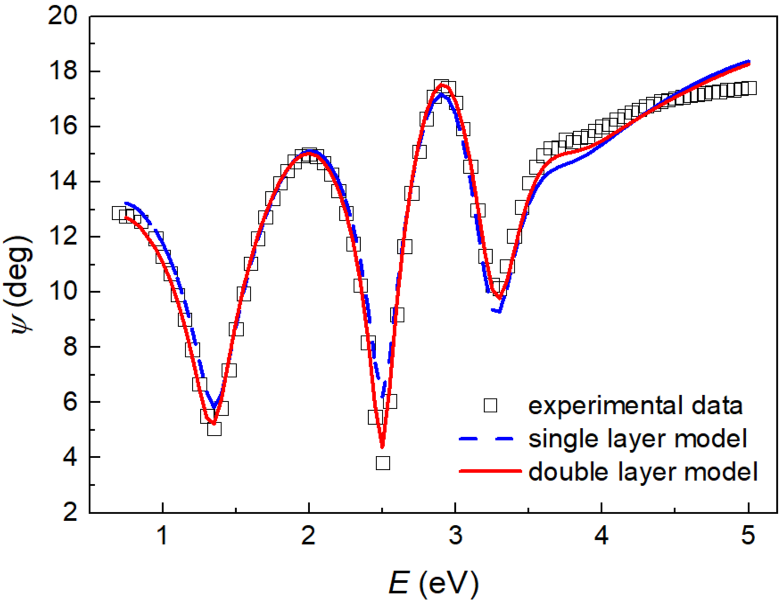

Fig. S3 Example of spectral dependence of ellipsometry parameter psi together two fits for angle of incidence 60 deg for double layered $Ge_{20}Sb_5Se_{75}$/$Ge_{20}Sb_5S_{75}$. Squares are measured data, blue dashed line is the best fit using model without intermediate layer (MSE ~ 11) and full red line is the best fit using model with intermediate layer (MSE ~ 4).

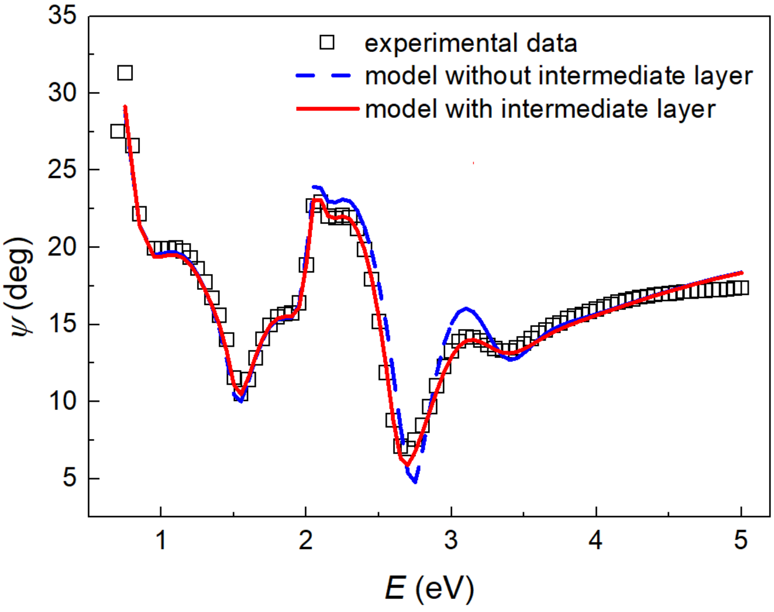

Fig. S4 Large area SEM scans (view field 1500x1500 µm) of hard-baked single- and multi-layered thin films.

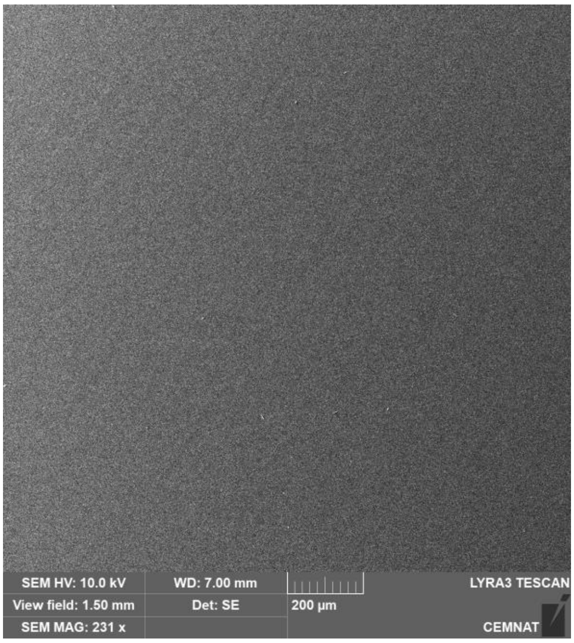

$Ge_{20}Sb_5S_{75}$ – single-layered thin film

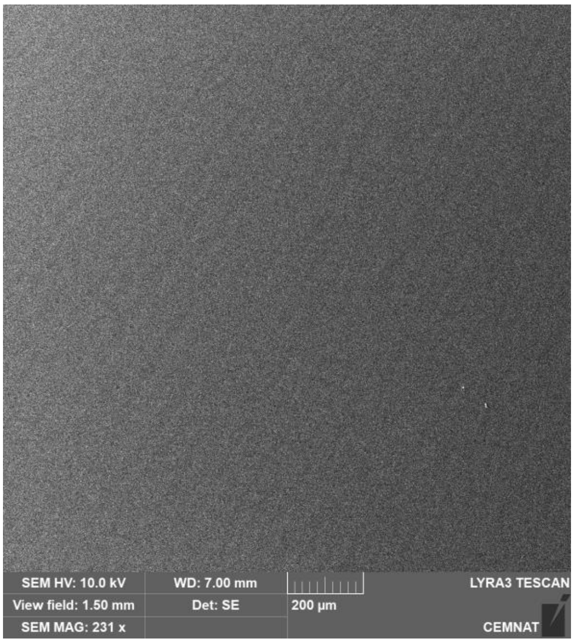

$Ge_{20}Sb_5Se_{75}$ single-layered thin film

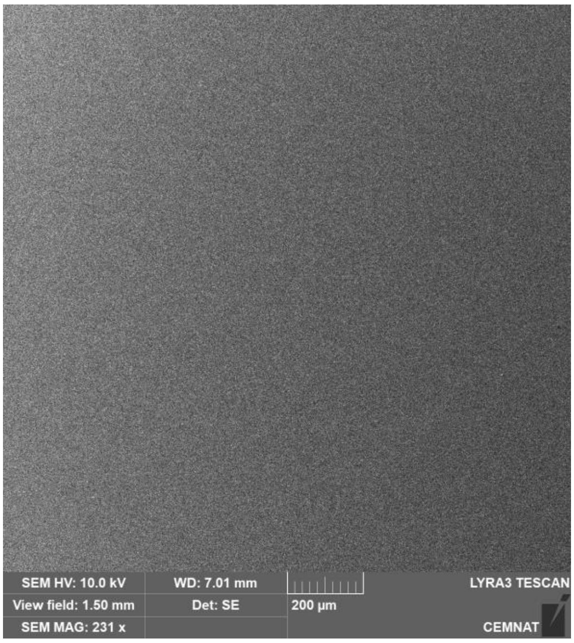

$Ge_{20}Sb_5S_{75}$ – double-layered thin film

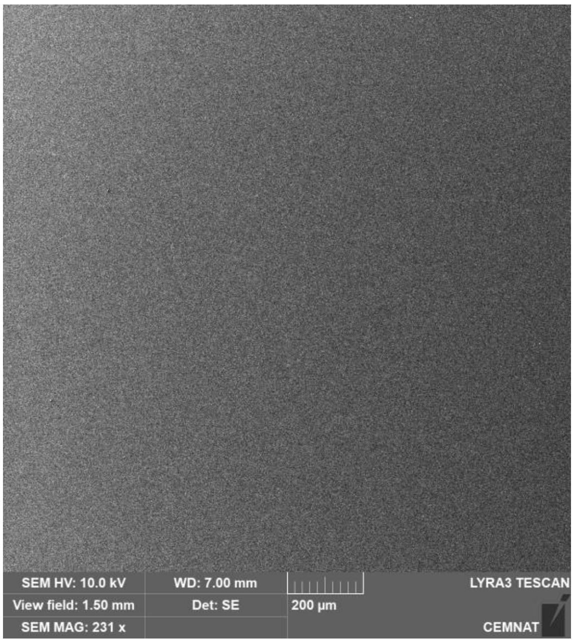

$Ge_{20}Sb_5Se_{75}$ – double-layered thin film

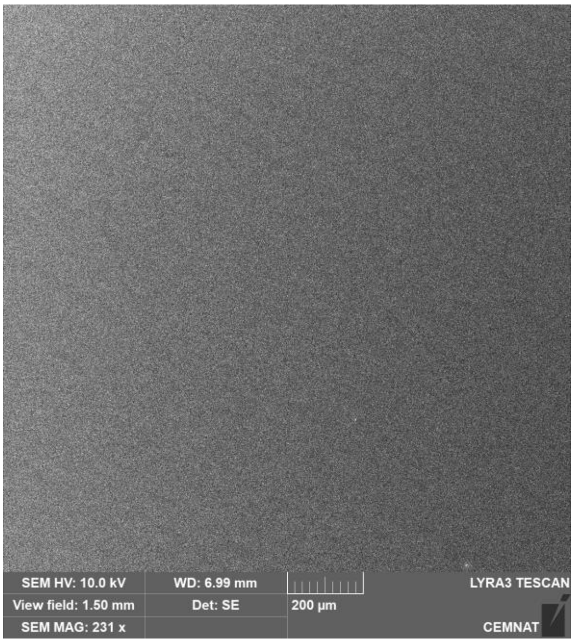

$Ge_{20}Sb_5S_{75}/Ge_{20}Sb_5Se_{75}$
double-layered thin film

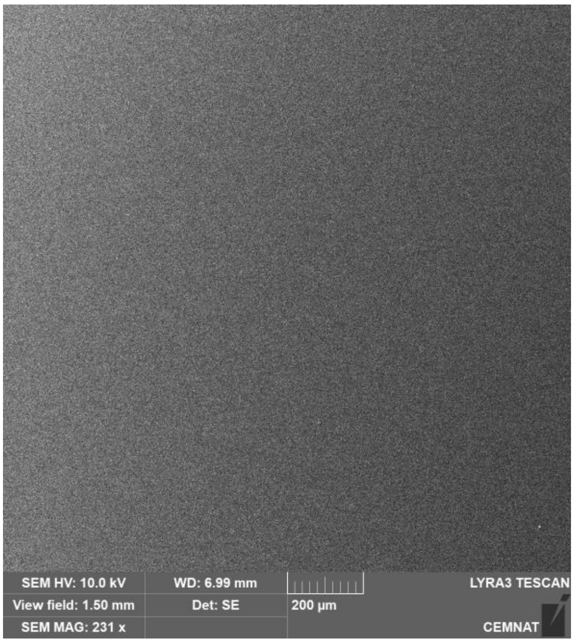

$Ge_{20}Sb_5Se_{75}/Ge_{20}Sb_5S_{75}$
double-layered thin film

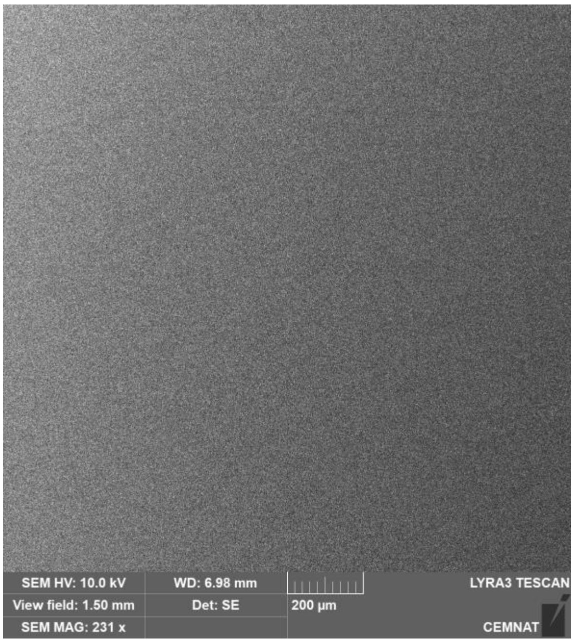

$Ge_{20}Sb_5S_{75}/Ge_{20}Sb_5Se_{75}/Ge_{20}Sb_5S_{75}$
triple-layered thin film

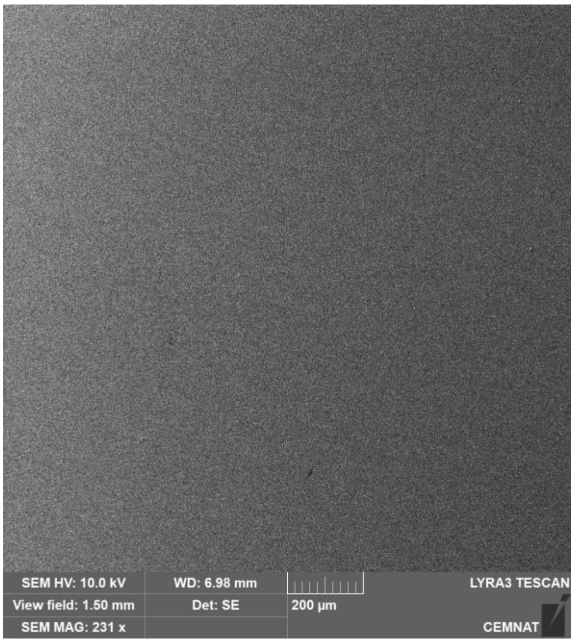

$Ge_{20}Sb_5Se_{75}/Ge_{20}Sb_5S_{75}/Ge_{20}Sb_5Se_{75}$
triple-layered thin film

Table S1 – Surface roughness values (RMS) of hard-baked single- and multi-layered thin films obtained by atomic force microscopy (AFM).

Sample	RMS (nm)
$Ge_{20}Sb_5S_{75}$ – single-layered thin film	0.93 ± 0.13
$Ge_{20}Sb_5Se_{75}$ – single-layered thin film	0.63 ± 0.14
$Ge_{20}Sb_5S_{75}$ – double-layered thin film	0.38 ± 0.06
$Ge_{20}Sb_5Se_{75}$ – double-layered thin film	0.31 ± 0.03
$Ge_{20}Sb_5S_{75}$/$Ge_{20}Sb_5Se_{75}$ double-layered thin film	0.30 ± 0.02
$Ge_{20}Sb_5Se_{75}$/$Ge_{20}Sb_5S_{75}$ double-layered thin film	0.25 ± 0.03
$Ge_{20}Sb_5S_{75}$/$Ge_{20}Sb_5Se_{75}$/$Ge_{20}Sb_5S_{75}$ triple-layered thin film	0.42 ± 0.16
$Ge_{20}Sb_5Se_{75}$/$Ge_{20}Sb_5S_{75}$/$Ge_{20}Sb_5Se_{75}$ triple-layered thin film	0.25 ± 0.04

Fig. S5 Atomic force microscopy (AFM) scans (field 5x5 μm) of hard-baked single- and multi-layered thin films.

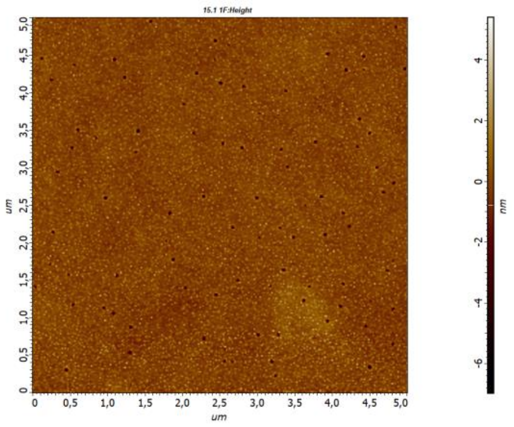

$Ge_{20}Sb_5S_{75}$ – single-layered thin film

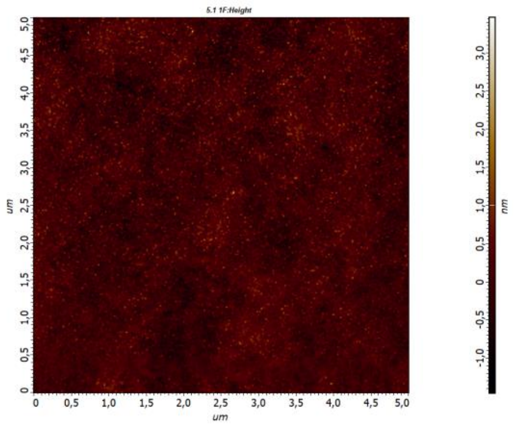

$Ge_{20}Sb_5Se_{75}$ single-layered thin film

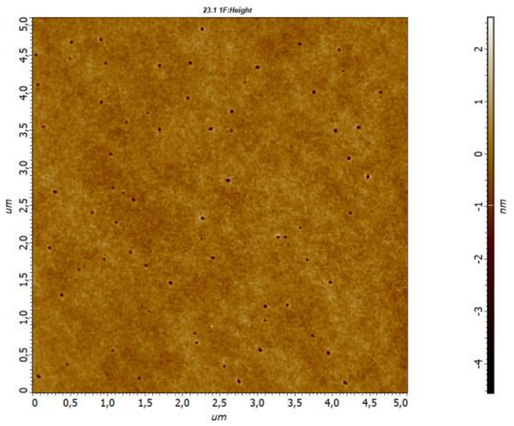

$Ge_{20}Sb_5S_{75}$ – double-layered thin film

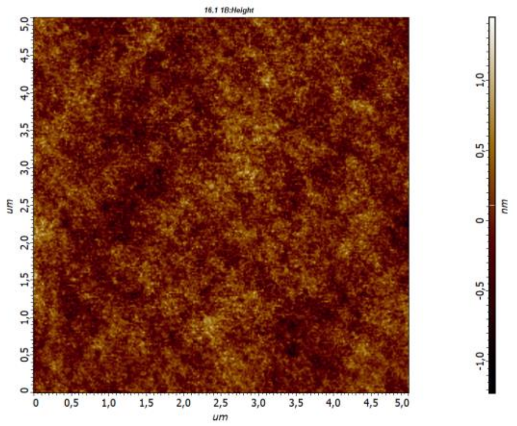

$Ge_{20}Sb_5Se_{75}$ – double-layered thin film

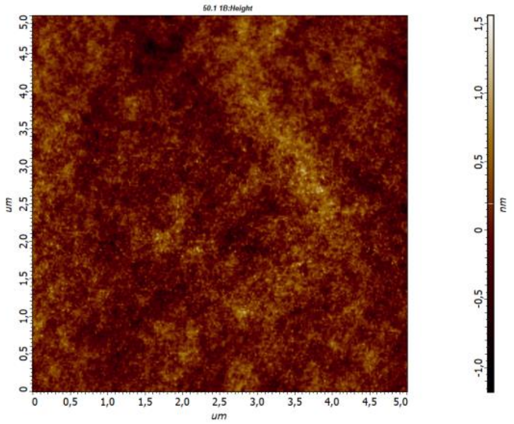

$Ge_{20}Sb_5S_{75}/Ge_{20}Sb_5Se_{75}$ double-layered thin film

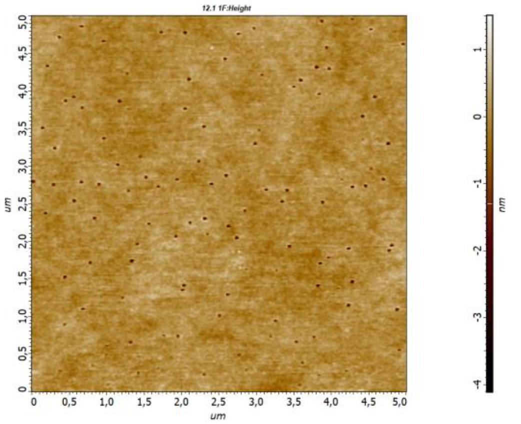

$Ge_{20}Sb_5Se_{75}/Ge_{20}Sb_5S_{75}$ double-layered thin film

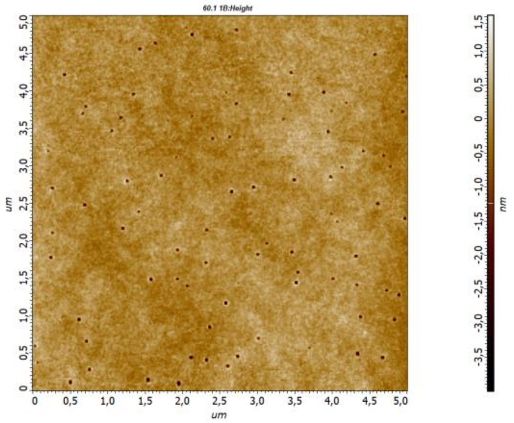

$Ge_{20}Sb_5S_{75}/Ge_{20}Sb_5Se_{75}/Ge_{20}Sb_5S_{75}$ triple-layered thin film

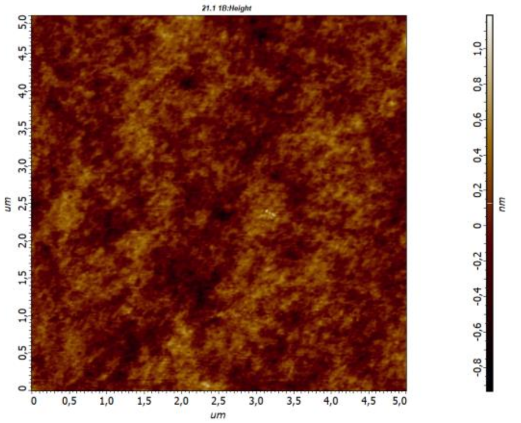

$Ge_{20}Sb_5Se_{75}/Ge_{20}Sb_5S_{75}/Ge_{20}Sb_5Se_{75}$ triple-layered thin film
